# Epidemiological Impact of Mandatory Vaccination against Hepatitis B in Italian Young Adults

**DOI:** 10.5812/kowsar.1735143X.723

**Published:** 2011-09-01

**Authors:** Marcello Campagna, Andrea Siddu, Angelo Meloni, Claudia Murru, Giuseppina Masia, Rosa Cristina Coppola

**Affiliations:** 1 Department of Public Health, University of Cagliari, Cagliari, Italy

**Keywords:** Hepatitis B, Vaccination, Effectiveness evaluation, Long-term immunity

## Abstract

**Background:**

Viral hepatitis caused by hepatitis B virus (HBV) is a leading cause of acute and chronic liver diseases worldwide.

**Objectives:**

In Italy, a mandatory vaccination policy was introduced in 1991 and was established for all newborns and 12-year-old individuals. In 2004, vaccination of 12-yearold adolescents was discontinued, and that of infants was maintained.

**Patients and Methods:**

We evaluated the seroprevalence of HBV markers in 806 individuals, who were vaccinated at birth or at 12 years of age, to assess the effectiveness of the national policy against HBV.

**Results:**

The overall prevalence of anti-HBs antibodies was 90.32% (95% confidence interval [CI]: 88.28–92.36%); 2.23% (95% CI: 1.21–3.25%) of the subjects were positive for both antibodies to HBsAg (anti-HBs) and antibodies to hepatitis B core antigen (anti-HBc), whereas 5.83% (95% CI 4.21–7.45) of the subjects were negative for all markers tested. Further, 1.61% (95% CI: 0.74–2.48%) of the subjects were positive for hepatitis B surface antigen (HBsAg).

**Conclusions:**

Our data provide additional evidence that HBV vaccination can confer long-term immunity when performed at birth and when performed for healthy adolescents; moreover, the results show the effectiveness of the application of a national vaccination strategy.

## 1. Background

Hepatitis B virus (HBV) contributes to most of the hepatitis disease burden worldwide [1]. After an acute infection, 1–10% of healthy adults and 30–90% of infected children become chronic carriers. WHO estimates that approximately 2 billion people worldwide have been infected by HBV, more than 350 million are chronically infected, and nearly 1 million people die every year from acute or chronic sequelae of primary infection with HBV [2]. The prevalence of HBV-related hepatitis varies across nations; in industrialized countries, the prevalence of this disease is less than 2% [3]. The epidemiology of HBV infection in Italy has changed substantially over the last few decades. According to the Acute Hepatitis National Surveillance System (SEIEVA), the incidence of acute HBV decreased from 12 per 100,000 in 1985 to 1.6 per 100,000 in 2008, with a consequent reduction of HBV-related chronic hepatic diseases from 50% to 13% [4]. This decrease in incidence was a consequence of the improvement of hygiene and socioeconomic conditions; campaigns for the prevention of the spread of HIV infection, which has the same route of transmission as HBV; introduction of the mandatory vaccination policy in 1991 for all newborns and 12-year-old adolescents; and mandatory screening for women in the third quarter of pregnancy [5].

In 2004, the vaccination of 12-year-old adolescents was discontinued, and that of infants was maintained. The objective of this vaccination strategy was to achieve a protection rate of 95% among the population in the age range of 0–24 years by 2003, thereby rendering the vaccination mandatory only for newborns and scheduling passive immunization within the first 24 hours of life; moreover, the strategy aimed to administer the first dose of vaccine to newborns whose mothers were seropositive for hepatitis B surface antigen (HBsAg) [6].

## 2. Objectives

The purpose of this study was to evaluate the seroprevalence of markers for HBV among a group of individuals submitted to the Italian vaccination strategy (at birth and at the age of 12 years, born between 1979 and 2010) to assess the effectiveness of the application of HBV vaccination strategy and the critical aspects and priorities of intervention.

## 3. Patients and Methods

The study population consisted of 806 individuals (372 male and 434 female individuals) admitted consecutively to the Diagnostic Service of the University Hospital of Cagliari– Monserrato. In this study, we enrolled all subjects in the 2 cohorts involved in the HBV national mandatory vaccination strategy; the first cohort comprised subjects who were born after 1991 and were vaccinated at birth (n = 57), and the second cohort comprised subjects who were born between 1979 and 1991 and were vaccinated atthe age of 12 years (n = 749); the median age of the subjects was 25.47 years. Enrolment was carried out between 2005 and 2010 to obtain a representative sample. The study protocol did not include a control group, becausevirtually, the entire Italian population that belonged to the same age group had been vaccinated [7]. All the 806 individuals enrolled in the study gave their written informed consent for participation; 0.86% (7/813) of the total number of the subjects recruited for the study did not agree to participate. Blood samples were obtained for all the subjects, and sera were collected and maintained at -20°C to measure the concentration of HBV serological markers (i.e., HBsAg, antibodies to HBsAg [anti-HBs], and antibodies to hepatitis B core antigen [anti-HBc]). We employed an immunoenzymatic commercial kit, AxSYM (Abbott).

Individuals with anti-HBs titers ≥ 10 mIU/mL were considered immunized by vaccination, whereas those with antibody titers < 10 mIU/mL were considered nonimmune. For those individuals who tested positive for HBsAg, a retrospective clinical history was collected to investigate the presence of risk factors and possible causes of infection. Differences in the frequency of hepatitis were detected by the Chi-Square test, and a P < 0.05 was considered significant. The 95% confidence interval (CI) for geometric mean titers and frequency of the disease was also calculated.

## 4. Results

**Figure 1:**
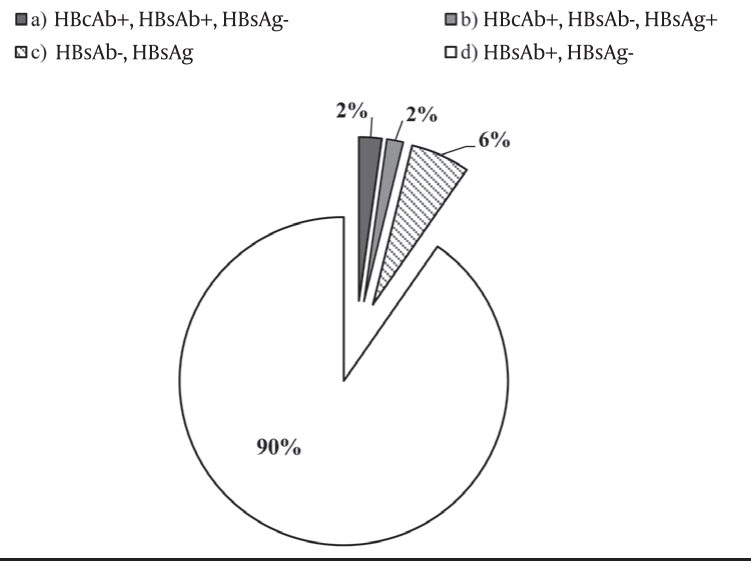
Prevalence of HBV Markers among the 806 Subjects.

Among the 806 individuals who were vaccinated either at birth or at the age of 12 years, the overall prevalence of anti-HBs antibodies was 90.32% (95% CI: 88.28–92.36). Moreover, 2.23% (95% CI: 1.21–3.25%) of the subjects were positive for both anti-HBs and anti-HBc antibodies (all of these subjects were from the 12-year-old cohort), whereas 5.83% (95% CI: 4.21–7.45) of the subjects were negative for all the markers tested. The rate of negative test results for all the markers was higher in the first cohort (12.5%) than in the second cohort (5.42%; Chi-Square test, P = 0.062). Positive test results for HBsAg were obtained for 1.61% (n = 13; 95% CI: 0.74–2.48) of the subjects (Figure 1). In the first cohort, 1 subject was positive for HBsAg; he was born to an HBsAg- and HIV-positive mother, and at birth, he was not submitted to an active-passive immunoprophylaxis policy established by the Italian legislation. In the second cohort, we found 12 subjects tested positive for HBsAg: 4 individuals were known to be HBsAg carriers at the time of vaccination proposal (3 were born to HBsAgpositive mothers; 1 was born in Romania, an endemic area for HBV infection, and was later adopted by Italian parents). Four individuals had been vaccinated and were subsequently identified as HBsAg carriers (1 lived with an HBsAg-positive mother, and the other 3 were not exposed to any known risk factors for HBV infection). One subject was tested positive for both HBsAg and HIV, but lacked further history pertaining to vaccination or risk factors; moreover, we could not obtain the clinical history of the other 3 individuals. Gender did not have an influence on the seroprevalence of anti-HBsAg antibodies (data not shown).

## 5. Discussion

The present study showed a high seroprevalence of anti-HBs antibodies owing to vaccination, with persistence of antibodies even beyond 10 years after vaccination. The proportion of individuals tested negative for anti-HBs antibodies observed in this study may be attributed to a loss of antibody concentration that can occur many years after vaccination; nevertheless, these individuals could be considered protected against HBV infection, as confirmed by recent scientific studies. Indeed, immunological memory for HBsAg is maintained even after the loss of anti-HBsAg antibodies, thereby providing effective protection against acute hepatitis and the development of the HBsAg carrier state [7][8][9]. These results confirm the efficacy of the mandatory Italian policy on immunization against HBV and its epidemiological impact, previously reported in 1998 with a coverage range at 24 months of age in Italy (85.6%–100%) and in our region (95.2%) [10]. Overall, the prevalence rate of anti-HBs antibodies is in agreement with the results of similar surveys [7]. The percentage of anti-HBs- and anti-HBc positive individuals suggests that an infection occurred after vaccination; however, this infection did not develop into a chronic disease owing to the vaccine-induced anti- HBsAg antibodies. Although our study was undertaken in a low endemicity country, these data along with the lower proportion of seronegative subjects in the second cohort than in the first cohort, despite the lack of significant difference between the 2 cohorts, suggest the possibility of a natural booster effect in vaccinated subjects in our region as well as in those living in regions of higher endemicity.

Our data show that in a substantial proportion of cases, HBsAg-positive individuals were already infected at the time of proposal of the vaccination policy. Seroconversion of individuals to HBsAg-positive individuals after vaccination is probably attributed to a pre-existing unknown infection at the time of vaccination and not necessarily to breakthrough infections. However, the fact that these individuals were included in the cohort implies the absence of vaccination at birth (because vaccination was not mandatory at that time) and inadequate vaccination because of their pre-existing unknown infections. Our study has some strengths and limitations; externalvalidity allows the comparison of the effectiveness of vaccination campaigns evaluated by studies in otherItalian regions as well as the identification of regional/ local differences in the application and effectiveness of Italian vaccination policies. The main limitation of our study is the collection of incomplete history for some individuals who were positive for HBsAg.

In conclusion, our data provide additional evidence that HBV vaccination can confer long-term immunity when performed at birth and also when performed for healthy adolescents [8][9][10]. Furthermore, this study showed the effectiveness and the need to apply the national vaccination campaign in the city of Cagliari. This study also highlighted the importance of a correct and exhaustive data collection procedure in a local surveillance system, to conduct surveys on the effectiveness of public health interventions. Data collection and analysis can also be useful to identify critical aspects, priorities, and further targeted interventions as well as to facilitate appropriate allocation of local health resources.
